# Transcriptomic reprogramming for neuronal age reversal

**DOI:** 10.1007/s00439-023-02529-1

**Published:** 2023-04-01

**Authors:** Alexandru M. Plesa, Michael Shadpour, Ed Boyden, George M. Church

**Affiliations:** 1 Department of Genetics, Harvard Medical School, Boston, MA USA; 2 Wyss Institute for Biologically Inspired Engineering at Harvard University, Boston, MA USA; 3 Department of Biological Engineering, MIT, Cambridge, MA USA; 4 Department of Brain and Cognitive Sciences, MIT, Cambridge, MA USA; 5 McGovern Institute for Brain Research, MIT, Cambridge, MA USA; 6 Howard Hughes Medical Institute, MIT, Cambridge, MA USA

## Abstract

Aging is a progressive multifaceted functional decline of a biological system. Chronic age-related conditions such as neurodegenerative diseases are leading causes of death worldwide, and they are becoming a pressing problem for our society. To address this global challenge, there is a need for novel, safe, and effective rejuvenation therapies aimed at reversing age-related phenotypes and improving human health. With gene expression being a key determinant of cell identity and function, and in light of recent studies reporting rejuvenation effects through genetic perturbations, we propose an age reversal strategy focused on reprogramming the cell transcriptome to a youthful state. To this end, we suggest using transcriptomic data from primary human cells to predict rejuvenation targets and develop high-throughput aging assays, which can be used in large perturbation screens. We propose neural cells as particularly relevant targets for rejuvenation due to substantial impact of neurodegeneration on human frailty. Of all cell types in the brain, we argue that glutamatergic neurons, neuronal stem cells, and oligodendrocytes represent the most impactful and tractable targets. Lastly, we provide experimental designs for anti-aging reprogramming screens that will likely enable the development of neuronal age reversal therapies, which hold promise for dramatically improving human health.

## The problem of aging

Aging is a major risk factor for multiple prevalent human diseases including cancer, cardiovascular disease, neurodegeneration, diabetes, and most recently COVID-19 (Fig. 1) (Xu et al. 2021; National Center for Health Statistics 2022). The mortality rate associated with all these diseases increases exponentially with age. Although deaths are the most apparent outcome of age-related diseases, the associated suffering and drop in quality of life also have a significant negative impact on our society (Netuveli et al. 2006). This is especially evident in the elderly suffering from dementia and neurodegenerative movement disorders, which reduce personal autonomy and independence and have no available cure.

**Figure:**
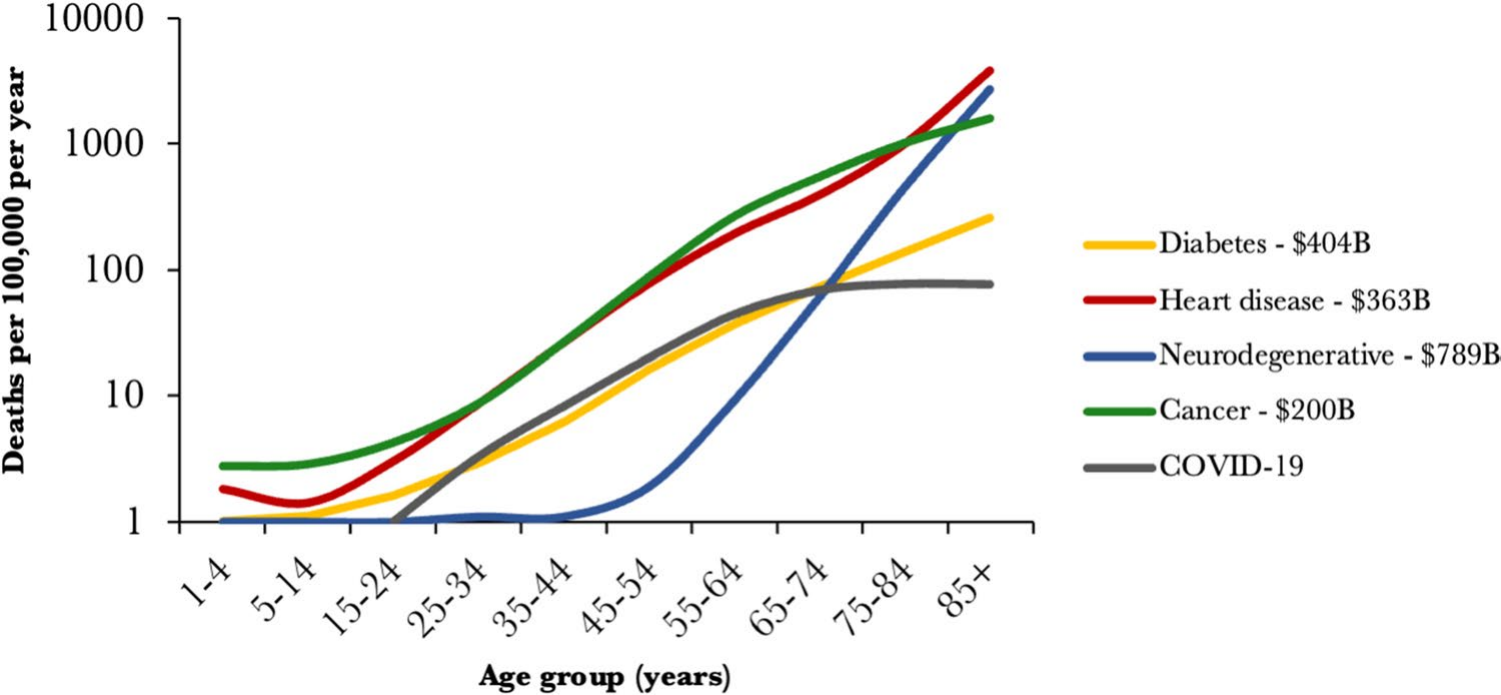

In addition to health implications, aging has a tremendous negative economic impact on our society. In the U.S., the population over 55 years old accounts for more than half the total healthcare spending (Jared et al. 2021), and most of these costs are associated with age-related diseases. For example, diabetes, heart disease, cancer, and neurodegenerative diseases have estimated associated healthcare costs of approximately $409B (2017) (O’Connell and Manson 2019), $363B (2017) (Virani et al. 2021), $200B (2020) (Mariotto et al. 2020), and $789B (2017) (Gooch et al. 2017), respectively. This does not include the impact of the recent COVID-19 pandemic on the U.S. economy, which is currently estimated at $363B (Office of the Actuary 2021) for 2020. Besides the these direct costs, aging also incurs indirect costs on our society through the associated drop in productivity and GDP growth. A recent study has modeled the economic value of targeting aging worldwide to approximately $38 trillion for an increase in life expectancy by just 1 year (Scott et al. 2021), highlighting the hidden costs of aging.

While the present social and economic challenges associated with aging are already pronounced, they are predicted to only increase in scale over the next decades. The United Nations has projected that the elderly population will more than double, from 9% to 23%, by the end of the century (United Nations 2019), exacerbating the negative effects of aging on our society. It is thus urgent and important to tackle the growing complex problem of aging, not only to avoid its effects on our economies, but also to increase the well-being of our growing elderly population.

## Aging and gene expression

The aging process is largely characterized by an age-dependent decline in physiological function that leads to an associated increase in all-cause mortality (Flatt 2012). In this article, we refer to aging as a progressive functional decline of a biological system, which allows the study of this process at different levels of abstraction. The most apparent of these levels is the organism, which, in humans, follows a universal life trajectory: development, stable organismal function, and progressive functional decline that ends in frailty and ultimately death. Interestingly, a similar trajectory is apparent at the tissue and cellular level, where the units of the systems slowly decay to a critical point, leading to various functional impairments.

One important aging dysfunction at the cellular level is that of gene expression, which is a central process under the direct regulation of epigenetics (Booth and Brunet 2016). Through the coordinated regulation of transcription, gene expression is the primary determinant of a cell’s structure and function. Accordingly, while all different cells of an organism share the same (unmutated) genome, their specification and function are dictated by the transcriptome. Therefore, the transcriptome is an evident layer that can be used to both read and write the state of a cell, including age related changes.

Indeed, several studies have thoroughly described the aging epigenome and transcriptome across species and cell types (Sen et al. 2016). Conserved age-related epigenetic changes include disruption of chromatin homeostasis characterized by histone loss (O’Sullivan et al. 2010), general genome-wide DNA hypomethylation (Pal and Tyler 2016), and chromatin remodeling due to altered histone modifications (Benayoun et al. 2015). These age-related changes tend to increase the entropy of the system (Tarkhov et al. 2022) by increasing variability in the epigenome (Hannum et al. 2013) and transcriptome (Sivakumar et al. 2022). For example, genomic regions marked by broad H3K4me3 domains were linked to increased transcriptional consistency, and perturbing the balance of this histone modification was shown to increase transcriptional variability (Benayoun et al. 2014). In line with these observations, Benayoun et al. (2019) have reported common age-related chromatin and transcriptional changes in the mouse heart, liver, and cerebellum that upregulated the innate immune response, with similar pathways also being affected in African turquoise killifish, rat, and human.

It is widely believed that the age-related loss of chromatin homeostasis leads directly to increased transcriptomic instability (Benayoun et al. 2015; Pal and Tyler 2016; Sen et al. 2016; Perez-Gomez et al. 2020), and a multitude of studies have confirmed this assertion in different biological systems. One of the first such studies was that of Bahar et al. (2006), which showed increased transcriptional noise in the aging mouse heart, as determined by the cell-to-cell variability in gene expression. Since then, with the advent of single-cell technologies, researchers have uncovered similar transcriptomic instability in mouse (Martinez-Jimenez et al. 2017) and human (Cheung et al. 2018) immune cells, human lung cells (Angelidis et al. 2019), and in the human pancreas (Gupta et al. 2021). Another related factor that increases the transcriptional noise with aging is the dysregulation of the splicing process, which is essential in maintaining the resilience and regulation of the transcriptome in response to different stimuli (Ferrucci et al. 2022). As such, splicing has been linked to aging and longevity, and due to its universal nature across species was proposed as a new hallmark of aging (Li et al. 2017; Bhadra et al. 2020).

A promising mechanistic link between the age-related transcriptional instability and the associated functional decline is the loss of cell identity, where the coordinated transcriptional program required for youthful function is affected by a downregulation of cell-type specific genes (Angarola and Anczuków 2021). In support of this paradigm, an age-related decrease in the expression of cell identity genes was initially observed in the human brain (Somel et al. 2010; Dönertaş et al. 2017; Soreq et al. 2017) and pancreas (Enge et al. 2017), and then confirmed in human skin (Solé-Boldo et al. 2019) and multiple mouse tissues (Salzer et al. 2018; Anisimova et al. 2020; Izgi et al. 2022). Moreover, transcript counts can integrate multiple layers of aging biology such as histone abundance and modification status (Yi and Kim 2020), chromatin organization and homeostasis, and even proteome diversity through isoform quantification. Taken together, these observations support a view of the transcriptome as a key cellular feature that can serve as a proxy for cell function in aging, making it an optimal target for assay development.

In line with this view, Peters et al. (2015) developed one of the first transcriptomic age predictors using microarray gene expression data from whole blood, which was able to predict donor age with a mean absolute error of 7.8 years. Using similar data, Mamoshina et al. (2018) trained a human muscle tissue transcriptomic clock with an mean absolute error of 6.2 years. With the advent of next generation sequencing, more recent transcriptomic clocks have used RNA-Seq data to build accurate age predictors for human T-cells (Remondini et al. 2017) and skin (Fleischer et al. 2018), as well as mouse lung (Choukrallah et al. 2020) and worms (Meyer and Schumacher 2020). Importantly, these transcriptomic clocks do not solely read out chronological age as they are able to report on features of biological aging, such as premature aging and longevity interventions.

## Age reversal

Age reversal is the process through which a subset or all of the features of an aged biological system are changed, irrespective of the path or trajectory, to resemble their youthful counterparts and impart a more functional state. Unlike slowing down the aging process, which can delay the age-related functional decline, the goal of age reversal is to improve functionality by recapitulating an earlier biological state.

While aging is a highly complex unsolved problem that spans multiple areas of biology, research has shown that a complete understanding of eukaryotic biology is not necessary for identifying interventions that can modulate the aging process. A landmark study that identified such a genetic perturbation is that of Kenyon et al. (1993), which showed that a single mutation in the *daf-2* gene can increase the lifespan of worms by more than 100%. This study sparked research interest in the biology of aging by demonstrating the malleability of the process through manipulation of specific genetic programs. Since then, there have been multiple studies showing the key roles of metabolism and growth pathways (e.g., insulin/IGF-1, mTOR, AMPK, and SIRT1) in longevity and lifespan (Kenyon 2010; Gems and Partridge 2013), thereby linking aging to the genomically encoded developmental program (Gladyshev 2016). However, until recently, it has been unclear whether aging is just malleable, or actually reversible.

Conceptually, the act of reversing the functional decline brought by the aging process is possible and takes place with each new life cycle. Through reproduction, a biologically aged germ cell, upon fertilization, gives rise to a young organism, thereby achieving age reversal at the species level. Rando and Chang (2012) used this observation to argue that, given the right signals, a similar age reversal could be achieved at the cellular level, via reprogramming of the epigenome to a youthful state. For example, Gurdon (1962) showed that, through somatic cell nuclear transfer (SCNT), adult differentiated nuclei can be transferred into enucleated oocytes and give rise to healthy frogs. Similar to the fertilization process, the cytoplasm of the egg reprograms the aged nuclei to reverse any accumulated age-related changes. This has been further demonstrated by the full and healthy lifespan of cloned sheep (Sinclair et al. 2016).

Gladyshev (2021) further explored this concept and proposed a “ground zero” of biological aging, whereby the early embryonic stages are associated with a universal rejuvenation process. This theory was recently tested and supported by applying multiple epigenetic aging clocks to mouse and human prenatal development, which revealed a rejuvenation period during early embryogenesis followed by the start of the aging process (Kerepesi et al. 2021). Following a similar development reversal path, the induction of the four Yamanaka factors (Oct4, Sox2, Klf4, and c-Myc, or OSKM) was shown to not only reprogram an aged somatic cell to an induced pluripotent stem cell (iPSC) capable of generating a newborn mouse (Takahashi and Yamanaka 2006; Zhao et al. 2010), but also reverse multiple features of aging such as cellular senescence, telomere size, oxidative stress, mitochondrial metabolism, and transcriptomic and epigenetic profiles (Lapasset et al. 2011; Petkovich et al. 2017). Moreover, several studies have shown beneficial rejuvenating effects through the transient induction of the Yamanaka factors in vivo (Ocampo et al. 2016; Kurita et al. 2018; Lu et al. 2020; Rodríguez-Matellán et al. 2020; Browder et al. 2022; Chondronasiou et al. 2022; Gill et al. 2022; Yang et al. 2023).

The mechanisms behind these age reversal paradigms are yet to be elucidated. Interestingly, it was shown that progenitor states are intrinsically more stable than their differentiated counterparts (Buganim et al. 2012; Stumpf et al. 2017), and a transient reversion to these states could potentially recover a youthful phenotype by replaying the developmental program. However, reprogramming cannot repair somatic mutations, which despite their relatively low frequency may contribute to aging by reducing the number of functional cells and increasing the likelihood of oncogenic transformation (Lodato et al. 2018; Cagan et al. 2022; Bae et al. 2022; Choudhury et al. 2022). Nevertheless, it is likely that the age reversal process can generate more fit and functional young cells while selecting against damaged cells that are beyond the point of rejuvenation. Such a potential mechanism is in agreement with the observed lower reprogramming efficiencies of old somatic cells compared to younger ones (Strässler et al. 2018). Moreover, this paradigm would parallel the reproductive age reversal strategy, whereby only a small fraction of aged gametes (potentially lacking major genomic and epigenomic alterations) can lead to successful embryos (Edwards 1999), thereby coupling genetic selection with cellular rejuvenation.

It is thus evident that age reversal is possible and has been achieved to varying degrees at both the species and cellular level, yet it remains elusive in vivo*.* The problem with fertilization, SCNT, and cellular reprogramming as approaches to age-reversal, is that they may pass through pluripotent developmental intermediates. This transition is either prohibitive at the organismal level or possesses a great teratogenic risk due to the loss of cell identity and acquisition of stem-like potential (Abad et al. 2013; Ohnishi et al. 2014; Ocampo et al. 2016; Senís et al. 2018). However, it has been proposed that it is possible to achieve age reversal without dedifferentiation (Manukyan and Singh 2012; Rando and Chang 2012; Olova et al. 2019), and a recent study showed there are several sets of factors that can perform cellular rejuvenation with varying degrees of cell identity loss (Roux et al. 2022). Therefore, a promising future direction for the aging field is the search for safe and effective interventions that can ultimately induce age reversal.

## Transcriptomic reprogramming

**Fig. 2:**
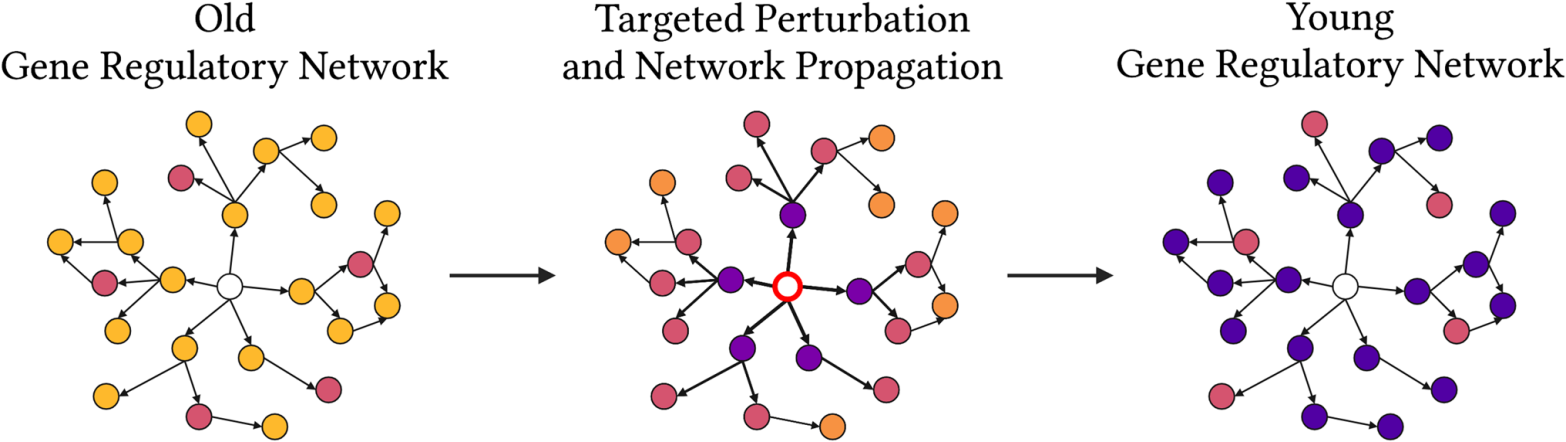
Schematic of transcriptomic reprogramming paradigm. An old gene regulatory network, where the gene expression of different members is dysregulated (yellow color), can be reprogrammed to a younger state (purple color) through the targeted perturbation of specific nodes (white color) and the subsequent network propagation of these rejuvenating effects

Transcriptomic reprogramming, defined as the transition between two defined transcriptomic states, is a promising method for age reversal. Using certain genetic perturbations, one can reprogram a specific gene regulatory network to a more youthful state and thereby rescue the function of the associated aged biological system (Fig. 2).

The permanence of a reprogrammed state is determined by the strength and duration of the initial perturbations, whose effect on key epigenetic regulators can in turn induce stable alterations in the chromatin landscape. For example, previous studies have shown that the efficiency of reprogramming fibroblasts to astrocytes is influenced by the dosage and frequency of mRNA delivery (Sul et al. 2009, 2012). While cellular reprogramming usually refers to the dedifferentiation of somatic cells to a pluripotent state through the use of the Yamanaka factors, transcriptomic reprogramming is applicable to any somatic or stem cell whose gene regulatory network is shifted towards a new steady state (Busskamp et al. 2014; Ng et al. 2021).

To identify these specific factors and protocols that can induce transcriptomic reprogramming to youthful states, we propose a three-step workflow inspired by the landmark transcriptomic aging screen performed by Janssens et al. (2019). First, transcriptomic data from differently aged primary cells are used to develop an aging assay and identify promising genetic targets for modulating the aging process. Second, these candidate genes are perturbed in vitro using high throughput gene modulating tools, and their effect on the aging phenotype is assayed using transcriptomic assays. Lastly, the most promising hits from the screen are validated for their rejuvenation potential using cell type specific functional assays in vitro and in vivo. Our recent experiments have offered a proof of concept for this pipeline by performing a transcriptomic reprogramming screen that identified SRSF1 overexpression as a rejuvenating intervention in primary dermal human fibroblasts (Plesa 2022).

## Neuronal age reversal

While rejuvenation of dermal fibroblasts offers an exciting proof of concept for transcriptomic reprogramming screens, there are other cell types of higher relevance for human disease. For example, reversing aging of neurons may prevent or delay cognitive decline, dementia, and neurological movement disorders. Understanding the mechanisms of age-related functional decline in neurons is increasingly important as the need to treat elderly patients with neurodegenerative disease increases (Hou et al. 2019).

In additional to clinical relevance, as post-mitotic cells that can live for decades, neurons present a unique and interesting model for studying aging. Firstly, damage that accumulates over time can be decoupled from that which emerges across cell divisions, like telomere attrition (Tomita et al. 2018). Additionally, as long-lived non-dividing cells (Bhardwaj et al. 2006), neurons face the unique challenge of maintaining genome and epigenome stability within each cell, with only certain brain regions having an active “backup” stem cell population (Ming and Song 2011; Ernst et al. 2014; Kase et al. 2020). Moreover, neurons perform such maintenance without the ability to dilute damage through cell division (Gladyshev 2014).

While primary neurons present important targets for age reversal due to their unique sensitivity to time-dependent dysfunction, they are especially difficult to maintain in culture (Kim et al. 2011). A popular alternative for modeling human neuronal aging is iPSC derived neurons (Dolmetsch and Geschwind 2011), ﻿which can﻿ be differentiated in large numbers from a single donor, allowing systematic characterization of different genetic backgrounds and screening of potential therapeutic targets. However, the reversion to an embryonic-like state through iPSC reprogramming can reset multiple hallmarks of aging (Lapasset et al. 2011; Horvath 2013; Petkovich et al. 2017). This drawback reduces the utility of such models for studying aging or age-related disease.

One potential solution is to artificially age iPSCs or iPSC-derived neurons in vitro. For example, some have modeled aspects of aging in iPSCs through telomerase inhibition (Vera et al. 2016). Neurons derived from these iPSCs have fewer dendrites, increased mitochondrial ROS, and increased rates of DNA damage. While these phenotypes overlap with the expected hallmarks of brain aging (Mattson and Arumugam 2018), the mechanism behind these dysfunctions fails to recapitulate in vivo biology. Importantly, telomere length of neurons in the human brain does not change significantly with age (Zhang et al. 2010; Tomita et al. 2018). In addition, mice with hyper-long telomeres (Muñoz-Lorente et al. 2019) do not display cognitive improvements or delayed cognitive decline. Others have modeled aging in vitro by overexpressing progerin (Miller et al. 2013), a truncated version of the lamin A protein, causally linked to Hutchinson-Gilford progeria syndrome (HGPS). In iPSC-derived fibroblasts, this overexpression phenocopies certain age-related cellular physiology including increased mitochondrial ROS and DNA damage, as well as a global loss of the heterochromatin protein 1 gamma (HP1γ), and tri-methylated H3K9 (H3K9me3). However, these age-related dysfunctions are only partially recapitulated in iPSC-derived neurons, which have no discernable heterochromatin changes upon overexpression of truncated lamin A. Furthermore, the HGPS model lacks a mechanistic explanation, as less than 1% of fibroblasts isolated from elderly donors express progerin (McClintock et al. 2007). While these methods show promise in more closely modeling aging phenotypes in neurons, they are not able to faithfully recapitulate the decades of wear and tear in vivo.

Another alternative system for studying brain aging is the transdifferentiation model, where a specific cell type is directly converted to neural fate, without going through a pluripotent intermediate. Importantly, transdifferentiation enables lineage conversion without the cellular age reset induced by dedifferentiation. For example, human primary fibroblasts can be converted directly to glial cells and neurons of various classes and subtypes (Xu et al. 2020). This conversion has been shown to maintain the transcriptomic and DNA methylation aging signatures (Mertens et al. 2015; Huh et al. 2016). Importantly, the integrated nature of the transcriptome in capturing aging information allows for maintenance of a detectable aging signature despite minimal overlap between the gene expression of transdifferentiated neurons and their fibroblast of origin.

The potential neuronal cell fates that can be attained by direct lineage conversion from fibroblasts and other somatic cells have been reviewed extensively elsewhere (Caiazzo et al. 2015; Mertens et al. 2018; Xu et al. 2020). While there is no consensus for which of these current possibilities should be prioritized for modeling brain aging, we consider glutamatergic neurons, neural stem cells (NSCs), and oligodendrocytes as the most promising in vitro models. Glutamatergic neurons are an attractive model system due to their high abundance in the human brain. Despite lacking an accurate estimation of neural cell type distribution (von Bartheld et al. 2016), recent studies place glutamatergic neurons as the most abundant human neural cell type, representing 40% of all neurons and up to 70% of all synapses in the brain (Victor Nadler 2010; Gasiorowska et al. 2021). Importantly, age related misregulation of glutamatergic activation leads to overactivity in the aged brain and this excitatory imbalance has been implicated in memory loss (Richardson et al. 2013). Restoring youthful activity levels in glutamatergic neurons via transcriptomic reprogramming holds promise for reversing age-related cognitive decline.

NSCs are another attractive target due to their experimental tractability, which stems from their ability to replicate in vivo and in vitro. This feature solves the problem of limited cell number that other transdifferentiated neurons suffer from, and it enables forms of functional genomic screening that are impossible in post-mitotic cells (Wu et al. 2022). However, as NSC transdifferentiation involves reversion of a somatic cell to a more progenitor-like state, it remains to be seen if this transition preserves age-related changes. On the other hand, the ability of NSCs to differentiate into multiple lineages enables straightforward characterization of a given genetic perturbation in multiple neural cell types (Louis et al. 2013; Vieira et al. 2018), including the biologically important glial cells (Rogister et al. 1999; Sher et al. 2008; Wang et al. 2018).

While neurons are the primary functional units of the brain, it is important to mention the relevance of glial cells for neuronal function and aging, especially since glia are the first cells in the human brain to exhibit a transcriptomic signature of aging (Rasband 2016; Jäkel and Dimou 2017; Sams 2021). It is likely that dysfunction of glial helper cells enables neural decline, analogous to T cell exhaustion enabling tumor growth (Zhang et al. 2020). Specifically, oligodendrocytes are the most abundant glial cell type (von Bartheld et al. 2016), thereby having the potential for systemic rejuvenation of the human brain. Oligodendrocytes are responsible for myelinating neurons, thereby providing insulation for efficient axon signaling, a process which becomes dysregulated in the aged brain (Tse and Herrup 2017; Sams 2021). Given that a single oligodendrocyte myelinates 20–60 axons (Simons and Nave 2016), rejuvenation of these cells in vivo could have an amplified impact on brain health. However, unlike neurons, oligodendrocytes are readily replaced by precursor cells and do not represent a long lived post mitotic population (Dawson 2003). They also lack the broad differentiation capacity of NSCs as well as their experimental tractability. Perhaps most importantly, the aging brain displays increased neural activity (Ghosh et al. 2021), therefore increased myelination and signaling efficiency could worsen this imbalance. For these reasons, while glial cells should not be ignored, a focus on neuronal age reversal is likely to yield stronger insights into improving aging brain health. However, as oligodendrocytes can be generated by direct conversion from fibroblasts, the approaches put forward in this article could also be applied to glial rejuvenation.

To identify genetic targets for transcriptomic reprogramming in neurons, the same approach as outlined above can be taken. First, a neuronal aging assay and perturbation candidate list can be generated from transcriptomic data. Second, primary somatic cells from donors of various ages can be engineered with inducible genetic perturbations and then transdifferentiated into the neural cell type of choice. The effects of these perturbations on the aging phenotype would then be assessed using high-throughput transcriptomic measurements. Lastly, in vitro and in vivo assays for functional rejuvenation, such as mitochondrial function and engraftment efficiency, can be used to validate promising targets.

One challenging aspect of this method is the limiting nature of neurons as post-mitotic cells. While all cell engineering and selection can be done before transdifferentiation, with post-mitotic cells only being induced and assayed for a phenotype, the number of somatic cells needed to generate enough neurons for scalable screening can be on the order of billions. Single cell perturbation screening techniques, such as crop seq and perturb seq (Dixit et al. 2016; Datlinger et al. 2017), offer a potential solution to this problem. By multiplexing inducible genetic perturbations in a pool of somatic cells before neural conversion, dozens of candidates could be screened using only thousands of cells. Even a genome scale screen of 20,000 perturbations, with each perturbation corresponding to 200 neurons, would be feasible and would require only 4 million cells. In addition to identifying age reversing gene candidates, the data generated by such a screen would create a first of its kind cell atlas of perturbed human neurons that can be used to gain new insights into fundamental aging and systems biology.

## Conclusion

There is a growing body of evidence for aging as the major risk factor for multiple chronic diseases, especially neurodegeneration. Considering the projected increase in the elderly population, aging is becoming a pressing problem for the world economy and wellbeing of our society, calling for increased scientific focus on rejuvenation interventions. Such research efforts have so far identified gene expression as a central regulator of the aging process. Furthermore, there have been several studies providing proof of concept data for cellular age reversal through genetic perturbations.

In this article, we build on the current knowledge base of aging and developmental biology to propose a new paradigm for identifying age-reversal genetic targets through perturbation based transcriptomic reprogramming screens. Using gene expression data from primary human cells, one can predict important aging regulators as well as develop high throughput assay for characterizing the aged cellular transcriptomic state. We argue that neurons serve as a compelling target for such approaches due to their essential role in neurodegenerative diseases, and we propose direct conversion of mature cells to induced neurons as a promising method for maintaining complex aging signatures in primary cells. Such a neuronal aging model provides a biologically relevant system for using high throughput perturbation screens to identify novel transcriptomic reprogramming factors for cellular rejuvenation. Ultimately, these factors will serve as targets for developing therapies to reverse age-related cognitive decline.
